# SRC Tyrosine Kinase Inhibitor and X-rays Combined Effect on Glioblastoma Cell Lines

**DOI:** 10.3390/ijms21113917

**Published:** 2020-05-30

**Authors:** Filippo Torrisi, Luigi Minafra, Francesco P. Cammarata, Gaetano Savoca, Marco Calvaruso, Nunzio Vicario, Laura Maccari, Elodie A. Pérès, Hayriye Özçelik, Myriam Bernaudin, Lorenzo Botta, Giorgio Russo, Rosalba Parenti, Samuel Valable

**Affiliations:** 1 Department of Biomedical and Biotechnological Sciences (BIOMETEC), University of Catania, 95123 Catania, Italy; filippo.torrisi@unict.it (F.T.); nunziovicario@unict.it (N.V.); 2 National Institute for Nuclear Physics, Laboratori Nazionali del Sud, INFN-LNS, 95123 Catania, Italy; luigi.minafra@ibfm.cnr.it (L.M.); savoca.gaetano@gmail.com (G.S.); marco.calvaruso@ibfm.cnr.it (M.C.); giorgio.russo@ibfm.cnr.it (G.R.); 3 Institute of Molecular Bioimaging and Physiology, National Research Council, IBFM-CNR, 90015 Cefalù, Italy; 4 Lead Discovery Siena s.r.l. (LDS), via Vittorio Alfieri, 31, Castelnuovo Berardenga, 53019 Siena, Italy; l.maccari@leaddiscoverysiena.it (L.M.); l.botta@leaddiscoverysiena.it (L.B.); 5 UNICAEN, CEA, CNRS, ISTCT/CERVOxy Group, GIP Cyceron, Normandie University, 14074 Caen, France; peres@cyceron.fr (E.A.P.); ozcelik@cyceron.fr (H.Ö.); bernaudin@cyceron.fr (M.B.); samuel.valable@cnrs.fr (S.V.)

**Keywords:** Glioblastoma, ionizing radiation, hypoxia, DNA damage, combined treatments

## Abstract

Glioblastoma (GBM) is one of the most lethal types of tumor due to its high recurrence level in spite of aggressive treatment regimens involving surgery, radiotherapy and chemotherapy. Hypoxia is a feature of GBM, involved in radioresistance, and is known to be at the origin of treatment failure. The aim of this work was to assess the therapeutic potential of a new targeted c-SRC inhibitor molecule, named Si306, in combination with X-rays on the human glioblastoma cell lines, comparing normoxia and hypoxia conditions. For this purpose, the dose modifying factor and oxygen enhancement ratio were calculated to evaluate the Si306 radiosensitizing effect. DNA damage and the repair capability were also studied from the kinetic of γ-H2AX immunodetection. Furthermore, motility processes being supposed to be triggered by hypoxia and irradiation, the role of c-SRC inhibition was also analyzed to evaluate the migration blockage by wound healing assay. Our results showed that inhibition of the c-SRC protein enhances the radiotherapy efficacy both in normoxic and hypoxic conditions. These data open new opportunities for GBM treatment combining radiotherapy with molecularly targeted drugs to overcome radioresistance.

## 1. Introduction

Radiotherapy (RT) represents a gold standard in the treatment of glioblastoma (GBM) that remains one of the most aggressive primary brain tumors with a high rate of recurrence [1]. Clinical data reported that RT is a positive prognostic factor on the survival of patients, as compared to patients that receive surgery or chemotherapy alone [2]. However, there are no clinical studies demonstrating an overall survival improvement with RT dosing above the standard of 60 Gy for 30 fractions (2 Gy/day), showing that there are two main issues to overcome: (i) Avoiding radiation side effects and (ii) reducing GBM radioresistance. The hypoxic pattern of GBM has been widely described and represents one of the main factors inducing radioresistance [3]. Hypoxic microenvironment reduces non-repairable DNA damage mediated by RT, as described by the hypothesis of oxygen fixation. Indeed, under normoxic conditions, molecular oxygen permanently fixes the DNA damage induced by free radicals produced in water radiolysis (indirect effects of ionizing radiation), being very genotoxic. Such a role, under hypoxic conditions, is proportionally reduced, thus affecting indirect damage induced by RT and establishing so called GBM radioresistance, leading to non-repairable DNA double strand breaks [4,5]. Therefore, hypoxic microenvironment, particularly pronounced in GBM, represents a poor prognosis factor, as shown both in preclinical models [6] and in human GBM patients [3,7]. Moreover, hypoxia mediates a favourable microenvironment to the growth and renewal of GBM stem cells and to the activation of specific proteins, involved in cell proliferation, angiogenesis, migration and invasion, that are the biological basis of GBM recurrence [8,9]. Among these proteins, SRC proto-oncogene non-receptor tyrosine kinase (c-SRC), a member of non-receptor SRC family kinases (SFKs), drives GBM carcinogenesis and progression, and is involved in intracellular signalling pathways related with hypoxia [10]. Several factors are involved in the activation of c-SRC, including focal adhesion kinase (FAK), integrins or tyrosine kinase growth factor receptors, like epidermal growth factor receptor (EGFR) [11]. Hypoxia stimulates the interaction of vIIIEGFR with the integrin β3 in GBM cells, activating a signalling pathways c-SRC-dependent resulting in the up-regulation of the cancer cell invasion markers, like matrix metalloproteinase-2 (MMP-2) and matrix metalloproteinase-9 (MMP-9) [12]. Therefore, c-SRC and its related network represent a key protein for targeted therapy.

Si306 (Lead Discovery Siena, Siena, Italy) is a molecule of the pyrazolo[3,4-d]pyrimidines family, which has been shown to inhibit c-SRC kinase protein activity [13]. Previous preclinical studies confirmed that Si306 was able to cross the intact blood–brain barrier and to progressively accumulate into the brain for 24 h after the post-intravenous injection. Moreover, it has been demonstrated that Si306 in combination with X-rays showed a synergic anti-proliferative effect in both in vitro and in vivo GBM models [14].

Herein, we aimed at investigating the Si306 capability to increase the radiotherapy efficacy both in normoxic and hypoxic conditions on the GBM cells, increasing the current knowledge on radiosensitizing effects of the novel c-SRC inhibitor Si306. For this purpose, we investigated the radiosensitizing effect of Si306 on two GBM cell lines, U251-MG and U87-MG, irradiated with X-rays in both normoxic (21% of oxygen) and hypoxic (1% of oxygen) conditions, and evaluated the degree of proliferation and migration. In addition, ɣH2AX foci detection by immunofluorescence was performed to quantify the radiation-induced DNA double-strand break formation and the DNA damage repair ability. Our results showed that c-SRC inhibition acted synergistically with radiation treatment, reducing clonogenic and migration ability and increasing DNA damage in GBM cells, in both normoxic and hypoxic conditions.

## 2. Results

### 2.1. c-SRC Inhibition Improves the Efficacy of Radiotherapy on U251-MG Cell Line

#### 2.1.1. Evaluation of Cell Survival from Clonogenic Assay

**Figure 1:**
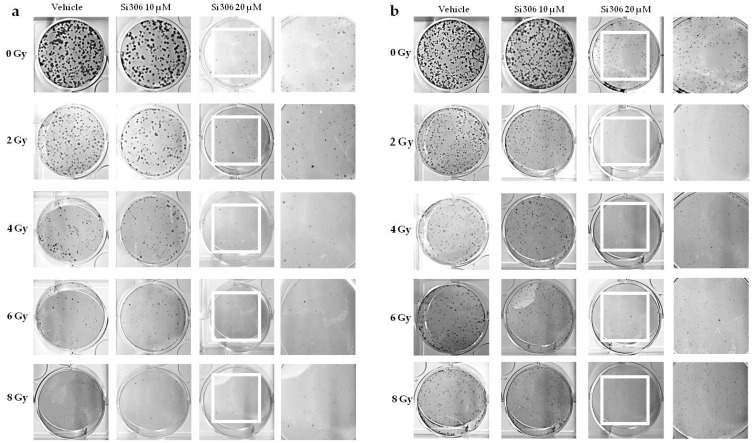
U251 clones after X-ray irradiation combined with Si306 in normoxia (21% oxygen) (**a**) and hypoxia (1% oxygen) (**b**).

**Figure 2:**
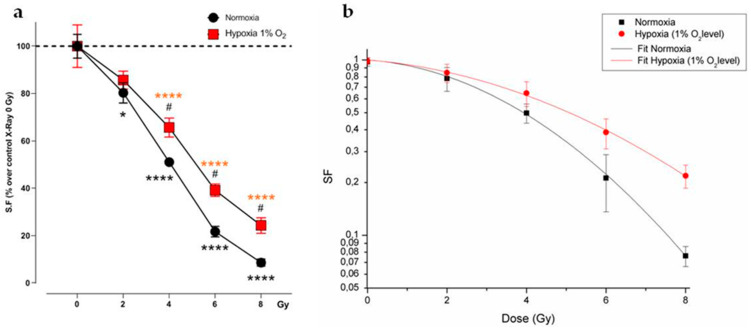
U251-MG irradiated cells in normoxia and hypoxia. (**a**) Surviving fraction (SF) plot of normoxic and hypoxic U251-MG cells exposed to 0, 2, 4, 6 and 8 Gy. Data are mean ± SEM of *n* = 3 independent experiments. * *p*-value < 0.05 and **** *p*-value < 0.0001 versus normoxia 0 Gy; # *p*-value < 0.05 versus each dose in normoxia (F_Si306conc._ = 133.8, *p*-value < 0.0001; F_Gy_ = 15.49, *p*-value = 0.0003; F_Si306conc. x Gy_ = 1.568, *p*-value = 0.1973. Two-way ANOVA with Holm–Šídák post-hoc test). (**b**) Linear-quadratic adjustment of the data of U251 cell survival curves treated with X-rays in hypoxia and normoxia.

**Figure 3:**
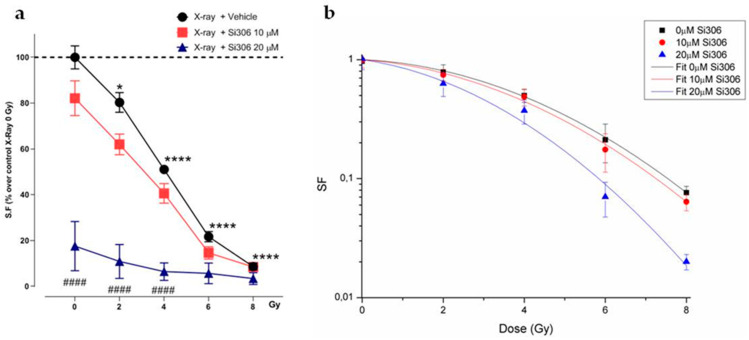
Cell survival of irradiated cells in normoxia with Si306 exposure. (**a**) SF plot of normoxic U251-MG cells exposed to 0, 2, 4, 6 and 8 Gy and treated with vehicle, 10 or 20 µM Si306. Data are mean ± SEM of *n* = 3 independent experiments. * *p*-value < 0.05 and **** *p*-value < 0.0001 versus 0 Gy in normoxia; #### *p*-value < 0.0001 versus only irradiated cells with 0, 2 and 4 Gy in normoxia (F_Si306conc_. = 89.17, *p*-value < 0.0001; F_Gy_ = 124.5, *p*-value < 0.0001; F_Si306conc. x Gy_ = 14.64, *p*-value < 0.0001. Two-way ANOVA with Holm–Šídák post-hoc test Two-way ANOVA with Holm–Šídák post-hoc test). (**b**) Linear-quadratic adjustment of the data of U251 cell survival curves treated with X-rays only and combined with Si306 in normoxia.

**Figure 4:**
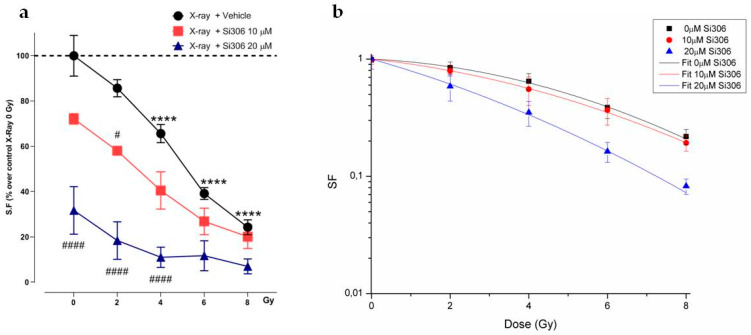
SF of irradiated cells with Si306 exposure in hypoxia. (**a**) Mean ± SEM, three independent experiments; **** *p*-value < 0.0001 versus 0 Gy in normoxia; # *p*-value < 0.05 and #### *p*-value < 0.0001 versus X-rays + vehicle at the same dose (F_Si306conc_. = 34.09, *p*-value < 0.0001; F_Gy_ = 77.95, *p*-value < 0.0001; F_Si306conc. x Gy_ = 3.929, *p*-value = 0.0012. Two-way ANOVA with Holm–Šídák post-hoc test). (**b**) Linear-quadratic adjustment of the data of U251 cell survival curves treated with X-rays only and combined with Si306 in hypoxia.

In order to compare the effects of increasing doses of X-rays (0, 2, 4, 6, 8 Gy) on U251-MG cell survival in normoxic (21% O_2_) and hypoxic (1% O_2_) conditions and in combination with 10 µM and 20 µM of the Si306 molecule, we performed clonogenic assays on the U251-MG cell line. The surviving fraction (SF) values were plotted against the dose to obtain dose-response curves. Dose modifying factor (DMF) and oxygen enhancement ratio (OER) were also calculated to evaluate treatment efficiency. The results showed a radiation dose dependent decrease in clone number with a significant effect with the exposition concomitant to Si306 (Figure 1a,b). Of note, U251-MG cells exhibited hypoxia-induced radioresistance with an OER of 1.27 (Figure 2a,b and Table 1). In normoxic conditions, the exposure to Si306 combined with RT induced a decrease in SF values with a DMF of 1.38 at the concentration of 20 µM (Figure 3a,b and Table 1). In hypoxic conditions, the effect of combined treatment was increased in culture exposed to Si306 versus control. The synergistic effect of Si306 and RT was further confirmed by the OER reduction of about 11%, demonstrating that c-SRC inhibition had a significant role as radiosensitizer in hypoxic conditions (Figure 4a,b and Table 1).

#### 2.1.2. Radiobiological Meaning of A, B and A/B Ratio Parameters

DMF and OER changes were also related to the α and β parameters analysis. These values displayed differences between groups (normoxia versus hypoxia) and treatment (vehicle versus Si306) (Table 2 and Table 3). The Si306 treatment combined with X-rays induced an α value increase in both conditions, in particular in the hypoxic one. Indeed, 10 and 20 µM Si306 showed α values of 0.092 ± 0.010 and 0.219 ± 0.025, respectively, as compared to control cultures (α value = 0.037 ± 0.024). This means that, in hypoxia, the linear contribution to damage is higher than in normoxia. The increase in β value is greater in normoxia rather than in hypoxia after exposure to Si306 in combination with irradiation, maybe due to ROS decrease in hypoxic condition. However, the DNA direct damage associated with α component may contribute to the OER decrease. Moreover, our data provided important evidence on the α/β value meaning that is an inverse reflection of a tissue sensitivity to dose fractionation. According to the α/β ratio, tissues are classified as early (low α/β) or late (high α/β) responding [15]. Therefore, the significant increase in the α/β ratio observed in hypoxia may represent a change in cellular radiobiological response leading to tissue patterns with a reduced ability to repair damage and with a greater accumulation of lethal lesions.

### 2.2. c-SRC Inhibition Sustains Radiation-Induced DNA Damage over Time 

**Figure 5:**
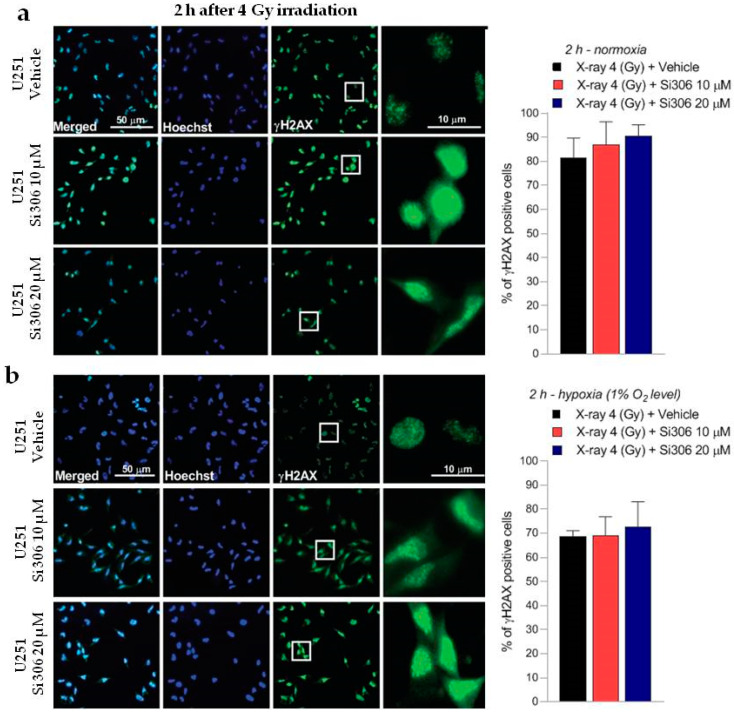
Representative pictures with inserts (white squares) and quantification of U251-MG positive cells for γH2AX performed 2 h after 4 Gy irradiation in normoxia (**a**) and hypoxia (**b**). Data are mean ± SD of *n* = 3 independent experiments; 4 Gy + vehicle; (F_normoxia_ = 2.030, *p*-value = 0.1564; F_hypoxia_ = 0.5685, *p*-value = 0.5798. One-way ANOVA with Holm–Šídák post-hoc test).

**Figure 6:**
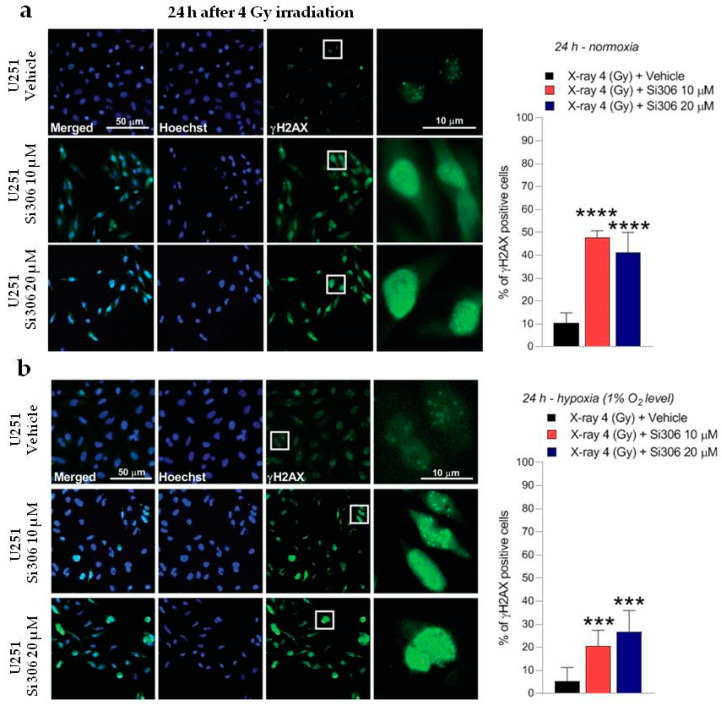
Representative pictures with inserts (white squares) and quantification of U251-MG positive cells for γH2AX realized 24 h after 4 Gy irradiation normoxia (**a**) and in hypoxia (**b**). Data are mean ± SD of *n* = 3 independent experiments; *** *p*-value < 0.001 and **** *p*-value < 0.0001 versus 4 Gy + vehicle (F_normoxia_ = 87.81, *p*-value < 0.0001; F_hypoxia_ = 18.87, *p*-value < 0.0001. One-way ANOVA with Holm–Šídák post-hoc test).

**Figure 7:**
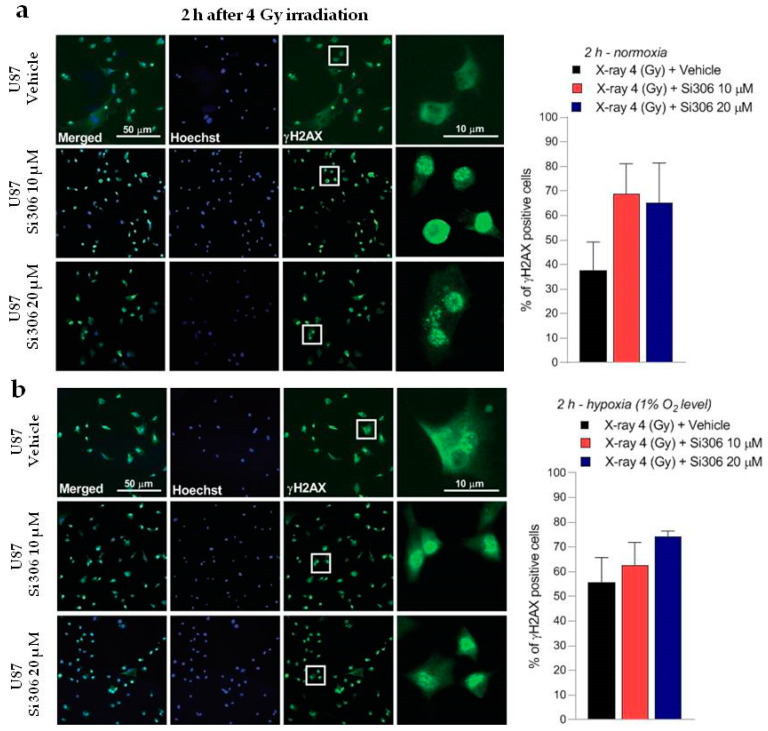
Representative pictures with inserts (white squares) and quantification of U87 positive cells for γH2AX performed 2 h after 4 Gy irradiation in normoxia (**a**) and hypoxia (**b**) Data are mean ± SD of *n* = 3 independent experiments (F_normoxia_ = 5.787, *p*-value < 0.0329; F_hypoxia_ = 4.048, *p*-value < 0.0557. One-way ANOVA with Holm–Šídák post-hoc test).

**Figure 8:**
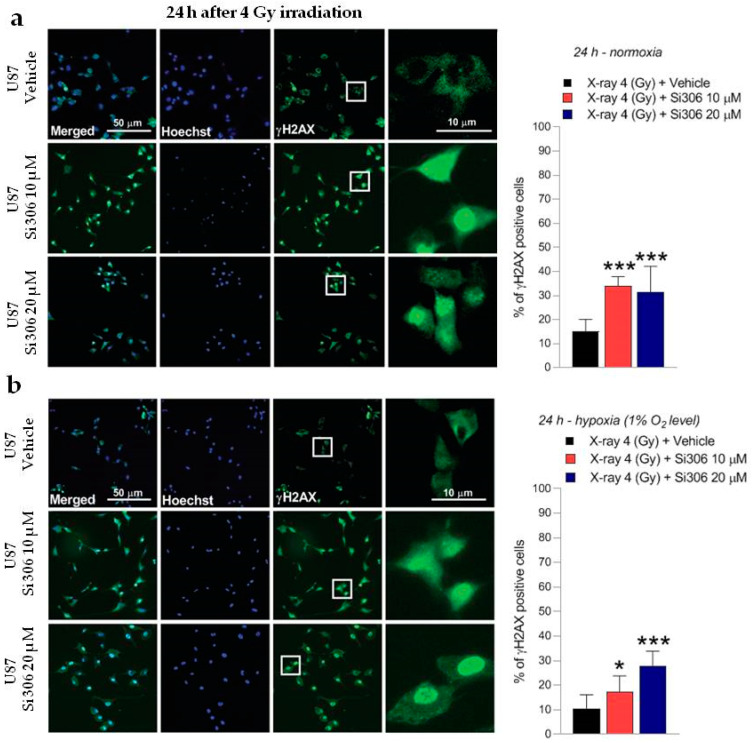
Representative pictures with inserts (white squares) and quantification of U87 positive cells for γH2AX realized 24 h after 4 Gy irradiation in normoxia (**a**) and hypoxia (**b**). Data are mean ± SD of *n* = 3 independent experiments; * *p*-value < 0.05 and *** *p*-value < 0.001 versus 4 Gy + vehicle (F_normoxia_ = 16.82, *p*-value < 0.0001; F_hypoxia_ = 12.77, *p*-value = 0.0004. One-way ANOVA with Holm–Šídák post-hoc test).

The DNA damage was evaluated by γH2AX immunofluorescence during the maximum of foci formation and also damage repair capacity (2 and 24 h after X-ray radiation, respectively) [9]. Immunofluorescence analyses showed that in normoxia and hypoxia, the exposure to Si306 in combination with irradiation led to a signal increase that was not significant 2 h after irradiation compared to X-rays only (Figure 5a,b). The synergistic effect of the Si306 molecule with IR became significant 24 h after treatment, where the foci signal was maintained at high levels in the case of combined treatment, compared to irradiation alone: 48 % and 41% of U251 cells, exposed, respectively, with 10 µM and 20 µM of Si306, were still positive compared to 10% of only irradiated U251 cells in normoxia. More interestingly, in hypoxia we showed a persistence of 21% and 27% positive U251-MG cells, both irradiated and exposed to 10 µM Si306 and 20 µM of Si306, respectively, compared to 5% of only irradiated cells (Figure 6a,b). To further confirm this observation, the immunofluorescence assay was repeated on the U87-MG GBM cell line. The results obtained were similar, since the differences in the foci γH2AX signal between the treatment conditions with vehicle and with Si306 were not significant 2 h after irradiation (Figure 7a,b). The increase in foci γH2AX expression was statistically significant only 24 h after irradiation in the combined treatments: In normoxia, after irradiation and Si306 pre-treatment, 35% (10 µM) and 31% (20 µM) of U87 cells were positive versus 15% of only irradiated U87 cells; similar results were obtained in hypoxia, since 18% and 28% of irradiated and Si306 pre-treated U87 cells, respectively, with 10 µM and 20 µM, were positive compared to 10% of only irradiated U87 cells (Figure 8a,b).

### 2.3. c-SRC Inhibition Reduces Cell Migration

**Figure 9:**
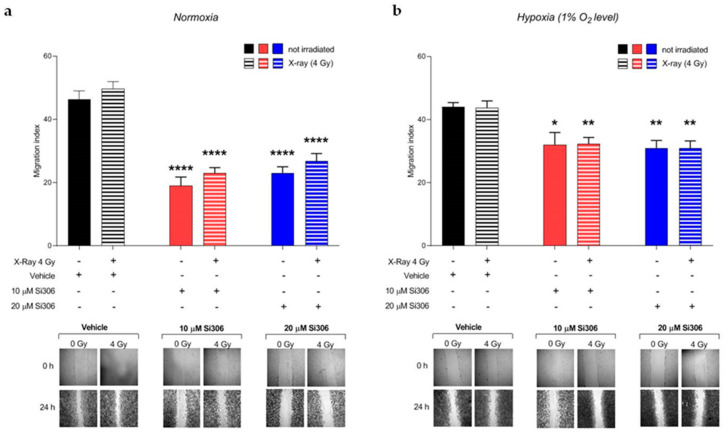
Effects of Si306 on migration of U251-MG cells in normoxia (**a**) and hypoxia (**b**). Data are mean ± SEM of *n* = 3 independent experiments. * *p*-value < 0.05, ** *p*-value < 0.01 and **** *p*-value < 0.0001 versus vehicle or vehicle + irradiation at 24 h after scratch (F_normoxia_ = 32.59, *p*-value < 0.0001; F_hypoxia_ = 6.907, *p*-value < 0.0001. One-way ANOVA with Holm–Šídák post-hoc test).

Migration and invasion of malignant glioma play a key role in GBM progression. Therefore, we examined, by wound healing assay, the effect of c-SRC inhibition on migration in irradiated U251-MG cells, being highly invasive, as reported in previous studies [16]. The results of wound healing assay showed an inhibitory effect of the Si306 molecule on the migration of the U251-MG cells. The addition of the Si306 molecule at both concentrations of 10 µM and 20 µM reduced the migration index of cells compared to those not irradiated and irradiated with a vehicle, in both normoxic and hypoxic conditions (Figure 9a,b).

## 3. Discussion

The poor prognosis of GBM represents an urgent clinical need and reinforces the necessity to explore and to develop novel therapeutic approaches. According to the clinical guidelines for the treatment of newly diagnosed GBM, only concomitant temozolomide with fractionated radiotherapy is indicated to significantly improve median survival (14.6 versus 12.1 months) and progression free survival (6.9 versus 5 months) as compared to RT alone, but high recurrences are still observed [17]. Therefore, specific cancer molecular targets are expected to have a synergistic effect to increase the efficacy of RT, overcoming radioresistance and modulating the irradiation dose delivered to enhance RT intrinsic sensitivity. During the last decade, molecular investigation on pathobiological mechanisms of GBM promoted research to develop molecularly targeted drugs (i.e., targeted therapy), including monoclonal antibodies (mAb) and tyrosine-kinase inhibitors (TKi), but their efficacy in the clinical practice is still limited as compared to conventional chemotherapy regimen [18]. c-SRC is a non-receptor tyrosine kinase (nRTK), interacting with many intracellular proteins, involved in GBM proliferation, invasion, motility and angiogenesis [10]. Previous evidence showed that hypoxia enhanced phosphorylation of tyrosine 416 in c-SRC, thus leading to protein-tyrosine kinase domain activation and to the downstream induction of VEGF expression, promoting angiogenesis [19]. Hypoxia may promote GBM progression and invasion throughout the integrin β3/FAK/SRC/EGFRvIII signalling axis, linking tumor cells and their surrounding environment [12]. Moreover, c-SRC activates HIF-1α and glucose uptake, thus fostering GBM proliferation rate [20].

Recently, we investigated Si306 molecule, a member of the pyrazolo[3,4-d] pyrimidines family, which is able to selectively bind and inactivate the ATP site of c-SRC protein, acting as ATP competitive inhibitor type I/II [13]. Combined approaches with X-rays irradiation showed that Si306 is able to reduce proliferation, survival and clonogenic ability of GBM cell lines, also promoting carcinoma-associated fibroblasts throughout TGFβ [14]. We previously showed that a combination of Si306 and proton irradiation holds great potential to induce synergic cytotoxic effects and modulate the complex gene network in in vitro models of GBM [21]. Given the pronounced hypoxia observed during GBM development and progression, we aimed at studying the role of the Si306 and X-ray combination in hypoxic conditions, generating dose/response curves and calculating OER in addition to DMF to evaluate the relationship of these two parameters.

We first confirmed that Si306 was able to reduce cell survival in normoxia and, importantly, whether such an effect was preserved in hypoxic conditions. Notably, clonogenic assay revealed that c-SRC inactivation had a significant impact in hypoxic cells, leading to a higher DMF and a lower OER. The α and β values also support these data, showing that the robust increase in the α/β ratio in hypoxic conditions was related to an increase in α value, thus indicating improved non-repairable DNA damage [22]. A potential explanation of such a significant effect of Si306 in hypoxic GBM cells may be related to the intrinsic biological response to low oxygen levels [23]. Hypoxia induces radioresistance promoting GBM invasion and activating specific intracellular machinery that also relies on c-SRC activation [12,24]. Previous studies showed that RT itself may positively relate to activation of invasion and migration mechanisms involving c-SRC proteins [25,26]. Our evidence suggests that Si306 contributes to reducing efficacy of endogenous self-protective mechanisms that took place in hypoxic conditions, particularly sensitizing cell populations relying on c-SRC activation [27]. Furthermore, the analysis of the γH2AX foci showed the c-SRC inhibition increases radiation-induced DNA damage and slows down the DNA repair abilities in both normoxic and hypoxic conditions. Importantly, Si306 treatment was also able to dramatically reduce cell migration in both normoxic and hypoxic conditions, thus indicating a substantial role of c-SRC pathway inhibition in GBM invasiveness.

Altogether, our data support the hypothesis that c-SRC inhibition may represent a promising approach to improve RT efficacy. Our evidences are in accordance with previous observations with the reference compound of c-SRC-family inhibitor PP2 [28] and with Si306 [14,29,30]. To date, the most important nTKI is the dual inhibitor c-SRC/Abl (Dasatinib) that was tested alone and in combination with mAb anti-VEGF (Bevacizumab), TKi of EGFR (Erlotinib) and alkylating agent (Lomustine) in clinical trials for recurrent GBM [31,32,33,34]. Results from randomized phase I/II trial of Dasatinib combined with Temozolomide and radiotherapy for newly diagnosed GBM does not show increased survival as compared to standard therapy alone [35]. The limitations of Dasatinib were associated to pharmacokinetics aspects due to efflux transporters P-glycoprotein, which are highly expressed in the blood–brain barrier and GBM cells [36]. On this aspect, recent evidence showed that Si306 hold higher cell growth inhibitory potential as compared to Dasatinib, and it was found to reduce P-gp activity in GBM cells with multidrug resistance phenotype in addition to an optimal brain penetration and accumulation on mice [37].

This work provided addition data supporting the benefit of c-SRC inhibition to enhance RT and, for the first time, investigated the efficacy of radiotherapy combined with c-SRC inhibition comparing normoxic and hypoxic conditions on GBM cell lines. Interestingly, our results indicated that Si306 molecule has a radiosensitizing effect on GBM cells both in normoxia and hypoxia, showing that it could be considered in a targeted strategy for GBM treatment.

## 4. Materials and Methods

### 4.1. Cell Culture and Hypoxia Experiments

The U251-MG and U87-MG human GBM cell lines were purchased from American Type Culture Collections (ATCC, Manassas, VA, USA) and cultured as previously described [21]. Cells were maintained in an exponentially growing culture condition, at 37 °C in a humidified atmosphere with 21% O_2_ and 5% CO_2_ (normoxic condition) and were subcultured in 75 cm^2^ standard tissue culture flasks. The U87-MG cells were used as additional cell line only for γ-H2AX immunofluorescence analyses.

For hypoxic experiments, 15 h after seeding, cells were transferred in the hypoxic workstation (IN VIVO2 1000, Ruskinn; Awel International, Blain, France), balanced with 94% N_2_ and 5% CO_2_ to maintain a gas concentration of 1% O_2_ at 37 °C (hypoxia). During experiment, cells were refilled with fresh medium previously equilibrated with the gas mixture containing 1% O_2_ in order to maintain this concentration from the beginning of the treatment with the drug.

### 4.2. Irradiation and Drug Treatments

Irradiation was performed in a biological irradiator (CellRad^®^, Faxitron, Edimex Le Plessis Grammoire, France) with a dose rate of 2 Gy/min, 130 kV and 5.0 mA. GBM cell irradiation was carried out using dose values of 2, 4, 6 and 8 Gy for clonogenic assay. 4 Gy dose was used for γ-H2AX immunofluorescence and migration assay.

The compound Si306 was provided by Lead Discovery Siena (Siena, Italy) as a stock powder and was dissolved in Dimethylsulfoxide (DMSO, Saint Quentin Fallavier, France). The Si306 molecule was diluted at a final concentration of 10 μM and 20 μM with fresh medium, in which GBM cells were maintained for 24 h. After irradiation, cells were replaced with fresh medium in order to remove the Si306 and manteined in normoxia or hypoxia up to the end of the experiment. The control samples for all biological tests were supplemented with vehicle (i.e., 0.5% DMSO).

### 4.3. Clonogenic Assay

Cells were seeded in a 6-well plates in triplicate at a density of 80–420 cells/cm^2^, according to the dose delivered and to the vehicle or drug concentration. Then, irradiation was performed using the dose values of 2, 4, 6 and 8 Gy. After irradiation, cells were incubated for 7–10 days in normoxia and hypoxia condition until the colony formation. The colonies were incubated with 0.05% crystal violet diluted in 20% ethanol (Saint Quentin Fallavier, France) for 30 min at room temperature. SF was determined according to the plating efficiency (PE) as we previously described [9]. Briefly, we calculated the PE, dividing the counted colony by the total plated cells. We then calculated the SF as a ratio of sample PE over control PE. For each experiment, the effect of each dose of radiation alone and combined with Si306 was evaluated on three individual wells of cell culture and each experiment was performed in triplicate.

### 4.4. Radiobiological Parameters Calculation

Surviving fraction values were adjusted according to the LQ model, which utilizes a multi-parameter equation for each individual experimental curve, the form of which is: S(D)/S(0)=e^(-αD-βD^2)^, where S(D) is the fraction of cells that survive at a given dose (D) and S(0) is the fraction of cells at 0 Gy; so we get α[Gy^−1^] and β[Gy^−2^] with their own standard deviation [21,38]. The DMF, which represents the dose of irradiation required to obtain the isoeffect, was calculated as previously described [21]. The OER, which is defined as the ratio of dose given under hypoxic conditions to the dose resulting in the same effect when given under normoxia [39], was also calculated. For both values of DMF and OER, the surviving fraction of 50% was considered a biological isoeffect at 0 µM, 10 µM and 20 µM of Si306.

### 4.5. γ-H2AX Immunofluorescence Analysis

Cells were seeded on sterile cover-glasses on 24 multiwell plates. After 8 h, cells were exposed to Si306 treatment for 24 h. Cells were then irradiated with 4 Gy and fixed in paraformaldehyde 4% at 2 and 24 h post-irradiation. Samples were then incubated with bovine serum albumin (BSA) 3% (Saint Quentin Fallavier, France), Tween 0.1% in PBS (Saint Quentin Fallavier, France) as blocking solution and to permeate cells for 30 min at room temperature. Indirect staining was performed using a primary antibody anti-γH2AX (1/1000; Abcam, ab26350, Paris, France) dissolved in BSA 1%, Tween 0.1% in PBS overnight at 4 °C. Then, samples were washed three times with Tween 0.1% in PBS for 5 min. Samples were incubated with Alexa-488-conjugated anti-mouse secondary antibody (1/500; Thermofisher Scientific, A-21202, Montigny Le Bretonneux, France) for 1 h. Nuclei were counterstained adding Hoechst 33342 stain (10 μg/mL; Saint Quentin Fallavier, France) for 1 h at room temperature. After three washes in PBS, samples were coverslipped and images were acquired using a Leica DM6000 microscope with a 20× objective. FITC and DAPI filter were used to detect foci γ-H2AX (in green) and nuclear signals (in blue), respectively. Quantifications were performed as previously described [40,41,42]. Briefly, images were analyzed using FIJI application software (version 2.0.0-rc-69/1.52p). Each region of interest was analyzed applying the iso-data threshold on immunofluorescence images of Hoechst and γ-H2AX and data are expressed as percentage of γ-H2AX positive nuclei over total Hoechst positive cells. Investigators blinded to the treatment groups performed all quantifications.

### 4.6. Migration Assay

Cells were seeded in 24 multiwell plates and incubated at both normoxic and hypoxic conditions. Following cell adhesion, Si306 molecule was added for 24 h. Mitomycin C (3 µL/mL, Saint Quentin Fallavier, France) was used to block cell proliferation. Samples were irradiated with 4 Gy, and immediately after the irradiation a horizontal scratch was created using a sterile tip in the center of the cell monolayer. After 24 h samples were washed with PBS to remove floating cells and were stained with crystal violet solution as mentioned above. Images were acquired at 0 hand 24 h post-scratch and the area between scratch edges was quantified. The scratch wound closure percentage was calculated as follows: The scratch area 0 h – the scratch area 24 h / (the scratch area 0 h) × 100%.

### 4.7. Statistical Analyses

All tests were performed in GraphPad Prism (version 5.00, GraphPad Software, San Diego, CA, USA). Data were tested for normality using a D’Agostino and Pearson omnibus normality test and subsequently assessed for homogeneity of variance. For comparison of *n* > 3 groups, one-way or two-way ANOVA was used where appropriate, followed by Holm–Šídák post-hoc test.

## 5. Conclusions

Further studies will help to better characterize the biological effects of Si306 in terms of cell toxicity and potential side effects. Taken together, the cell survival reduction, supported by DMF and LQ model, the DNA damage increase and the migration inhibition are all effects induced by the combination of a Si306 molecule and X-rays in both conditions of normoxia and hypoxia. For this reason, Si306 is a potential candidate as a new radiosensitizer in targeted therapy to overcome radioresistance in GBM disease.

## Figures and Tables

**Table 1:** Dose modifying factor (DMF) and oxygen enhancement ratio (OER) values calculated as isoeffective dose at surviving fraction of 0.5.

Treatment	Normoxia SF50% (Gy)	Hypoxia SF50% (Gy)	Normoxia DMF	Hypoxia DMF	OER
X-rays + vehicle	4.09	5.18	1	1	1.27
X-rays + 10 μM Si306	3.86	4.53	1.05	1.15	1.17
X-rays+ 20 μM Si306	2.54	2.67	1.38	1.94	1.05

**Table 2:** α and β parameters by fitting the cell survival to the linear-quadratic (LQ) model in normoxia. Values correspond to mean ± SEM; three independent experiments.

Treatment Normoxia	α (Gy-1)	β (Gy-2)	α/β (Gy)
X-rays + vehicle	0.037 ± 0.011	0.036 ± 0.009	1.03
X-ray s+ 10 μM Si306	0.060 ± 0.039	0.035 ± 0.009	1.71
X-rays+ 20 μM Si306	0.077 ± 0.009	0.052 ± 0.005	1.48

**Table 3:** α and β parameters estimated by fitting the cell survival to the linear-quadratic in normoxia (LQ) model in hypoxia. Values correspond to mean ± SEM; three independent experiments.

Treatment Hypoxia	α (Gy-1)	β (Gy-2)	α/β (Gy)
X-rays + vehicle	0.037 ± 0.024	0.020 ± 0.005	1.85
X-rays + 10 μM Si306	0.092 ± 0.010	0.013 ± 0.002	7.07
X-rays + 20 μM Si306	0.219 ± 0.025	0.014 ± 0.005	15.64

## Abbreviations

**G**BM	**G**lioblastoma
RT	Radiotherapy
MMP-2	Matrix metalloproteinase-2
MMP-9	Matrix metalloproteinase-9
SFKs	SRC family kinases
FAK	Focal adhesion kinase
EGFR	Epidermal growth factor receptor
SF	Surviving fraction
PE	Plating efficiency
DMF	Dose modifying factor
OER	Oxygen enhancement ratio
LQ	Linear-quadratic
mAb	Monoclonal antibodies
TKi	Tyrosine-kinase inhibitors
nRTK	Non receptor tyrosine kinase
ECM	Extracellular matrix
DMSO	Dimethylsulfoxide
BSA	Bovine serum albumin
